# Correlation between TSP-1, TGF-β and PPAR-γ expression levels and glioma microvascular density

**DOI:** 10.3892/ol.2013.1650

**Published:** 2013-10-29

**Authors:** JING ZHANG, WEI YANG, DUANYUN ZHAO, YUN HAN, BO LIU, HUA ZHAO, HONGBO WANG, QUANZHONG ZHANG, GUANGMING XU

**Affiliations:** 1 Department of Neurosurgery, Shandong University, Jinan, Shandong 250100, P.R. China; 2 Department of Neurosurgery, Shandong Provincial Hospital, Jinan, Shandong 250014, P.R. China; 3 Department of Neurosurgery, Heze Municipal Hospital, Heze, Shandong 274000, P.R. China

**Keywords:** thrombospondin-1, transforming growth factor-β, peroxisome proliferator-activated receptor-γ, microvessel density, glioma

## Abstract

Gliomas are the most common type of primary tumor in the central nervous system and are characterized by abundant capillary angiogenesis. It is important to study the underlying molecular mechanisms of angiogenesis in order to aid the identification of potential therapeutic targets. The aim of the current study was to investigate the expression levels of thrombospondin-1 (TSP-1), transforming growth factor-β (TGF-β) and peroxisome proliferator-activated receptor-γ (PPAR-γ) in gliomas, and determine their relationships with angiogenesis. Immunohistochemical methods were used to detect TSP-1, TGF-β and PPAR-γ expression levels and to assess microvascular density (MVD) in 99 glioma tissue samples of various grades. The total positive expression rates of TSP-1 and PPAR-γ were 78.4 and 94.1% in low-grade gliomas and 45.8 and 39.6% in high-grade gliomas. These values suggest that their expression negatively correlated with tumor grade. However, TGF-β expression positively correlated with tumor grade; the total positive expression rate of TGF-β in high-grade gliomas (93.8%) was significantly increased compared with that in low-grade gliomas (43.1%). The MVD in the low-grade group was 28±7.2 vessels/field, which was significantly lower than in the high-grade group (45±6.2 vessels/field). TSP-1 and PPAR-γ expression levels were negatively correlated with MVD (P<0.05), while the TGF-β expression level was positively correlated with MVD (P<0.05). These results indicate that the TSP-1, TGF-β and PPAR-γ expression levels in gliomas are correlated with MVD, which suggests that these proteins may be involved in the regulation of glioma angiogenesis.

## Introduction

Malignant gliomas are the most common type of primary tumor in the central nervous system. Not only are they very difficult to treat, their invasive nature makes recurrence likely (1). Gliomas are typical vascular-dependent solid tumors that induce an abundance of new capillaries, which provides the structural basis for rapid tumor cell proliferation, invasion and recurrence (2). The process of angiogenesis is tightly regulated by multiple factors; thus, understanding the molecular underpinnings of angiogenesis and how to effectively inhibit this process is critical for the treatment of malignant gliomas (3,4).

Thrombospondin-1 (TSP-1) is an extracellular matrix glycoprotein that contains multiple functional domains. It is involved in the proliferation and adhesion of tumor and epithelial cells. TSP-1 also strongly inhibits tumor angiogenesis, thus inhibiting malignant tumor growth and metastasis (5,6).

Transforming growth factor-β (TGF-β) is important in promoting tumor progression and is expressed in most cell and tissue types. Previous studies have demonstrated that TSP-1 activates TGF-β precursor, thus increasing TGF-β expression levels in tumor tissues, which in turn promotes tumor angiogenesis, growth and invasion (1–4).

Peroxisome proliferator-activated receptor-γ (PPAR-γ) is the most notable factor associated with TGF-β. It is a member of the nuclear receptor superfamily that is able to inhibit malignant tumor progression following activation via its ligands. Previous studies have identified that PPAR-γ agonists are capable of inhibiting TGF-β-induced metastasis in lung cancer (7). Furthermore, PPAR-γ is involved in adipocyte differentiation, insulin resistance, glucose metabolism, inflammation, the immune response and tumorigenesis (8,9).

The relationships between TSP-1, TGF-β and PPAR-γ expression levels and microvascular density (MVD) in gliomas are unknown. The present study examined the expression levels of these three proteins in different grades of glioma using immunohistochemical staining, and investigated their relationships with MVD.

## Materials and methods

### Clinical data and reagents

From June, 2011 to July, 2012, a series of 99 patients with pathologically confirmed gliomas, who were seen in the Department of Neurosurgery, Provincial Hospital Affiliated to Shandong University (Jinan, China), were studied. All patients were undergoing a first surgery and had not received pre-operative radiation or chemotherapy. The subject pool was comprised of 54 males and 45 females aged 6–71 years (median age, 47±1.2). The tumor tissues were fixed in 10% neutral formalin prior to being embedded in paraffin. Grading of the tumors according to the 2007 WHO classification of tumors of the central nervous system (10), indicated that there were 21 grade I astrocytoma cases, 30 cases of grade II astrocytoma, 29 cases of grade III anaplastic astrocytoma and 19 cases of grade IV anaplastic astrocytoma and glioblastoma. Fifty-one cases were in the low-grade group (I–II) and 48 cases were in the high-grade group (III–IV; Table I). Reagents used in this study included; rabbit anti-human TSP-1 polyclonal antibody and PPAR-γ rabbit anti-human polyclonal antibody (Beijing Biosynthesis Biotechnology Co., Ltd., China); rabbit anti-human TGF-β polyclonal antibody (Abcam, Cambridge, MA, USA); CD34 rabbit anti-human polyclonal antibody (Wuhan Boster Biotechnology Co., Ltd., China); and secondary antibodies from an immunohistochemistry kit for rabbit antibodies (Beijing Zhongshan Golden Bridge Biotechnology Co., Ltd., China). All experiments were approved by the Ethics Committee of Shandong University School of Medicine (Jinan, China). The specimens used in this study were approved by the Provincial Hospital Affiliated to Shandong University (Jinan, China), and all patients provided written informed consent prior to enrollment.

### Methods

#### Immunohistochemistry [streptavidin-biotin complex (SABC) method]

Paraffin-embedded glioma specimens were sliced into 4-μm thick sections, which were deparaffinized in turpentine, rehydrated in an ascending ethanol series, and incubated in a citrate buffer (0.01 mol/l, pH 6.0) with water bath heating (~98ºC) for 15 min for antigen recovery. Subsequently, the sections were rinsed with phosphate-buffered saline (PBS) for 5 min prior to the dropwise addition of normal goat serum blocking solution. Following incubation at room temperature for 20 min, the sections were treated with the primary antibody dropwise, incubated at 37ºC for 1 h, and then rinsed with PBS buffer three times (2 min each). Thereafter, the sections were treated with anti-rabbit biotinylated secondary antibody, incubated at 20–37ºC for 20 min, and then rinsed with PBS buffer three times (2 min each). Subsequently, the sections were treated by the dropwise addition of SABC, incubated at 20–37ºC for 20 min, and rinsed with PBS buffer four times (5 min each). Following a general SABC immunohistochemical protocol, immunoreactivity was visualized with 3,3′-diaminobenzidine and the nuclei were counterstained with hematoxylin. Sections were subsequently dehydrated in a series of graded alcohols, cleared in xylene and coverslipped. Breast cancer samples from the Department of Pathology, Provincial Hospital Affiliated to Shandong University were used as the positive control and slides incubated with PBS without antibody were used as the negative control.

#### Immunohistochemical scores

The presence of brown or yellow-brown granular cytoplasmic or intracellular staining was considered as positive staining. Cells were counted in five randomly selected fields (magnification, ×200) and the percentage of positive cells was calculated. According to semi-quantitative immunohistochemical methods, the staining density and range were evaluated by integrated scoring. The staining density of the cells and the number of positive cells were scored as 0–3, and the staining density was determined according to the degree of coloration in the majority of cells. The staining density of cells was scored as follows: Cytoplasm or intercellular substances with a light brown or light yellow color, 1; brown or yellow color, 2; deep brown or deep yellow color, 3; and non-stained, 0. The number of positive cells (percentage) was scored as follows: <25%, 1; 26–50%, 2; >50%, 3; and no cell staining, 0. According to the integration of the above two indicators, four levels were graded as follows: 0, negative (−); 2 or 3, mild positive (+); 4, positive (++); and 5 or 6, intense positive (+++).

#### MVD evaluation

CD34 was used as a marker of vascular endothelial cells to indicate blood vessels. The three most vascularized areas within each tumor were viewed at low magnification (×100), and intratumoral vessels were imaged and counted in five fields at ×400 magnification by a blinded investigator. Any single cell or cell cluster stained by the antibody, regardless of its formation of lumen, was taken as a countable microvessel if it formed a clear separation with the surrounding vessels, tumor cells and other tissues. The mean value of the quantified images from the three regions was considered as the MVD (Table II).

#### Statistical analysis

Statistical analysis was performed with SPSS, version 18.0 software (SPSS, Inc., Chicago, IL, USA). Data are expressed as the means ± SD. Between-group comparisons of low- and high-grade gliomas were performed with χ^2^ tests. Within-group changes of low- and high-grade gliomas were analyzed using Student’s t-tests. The correlations between TSP-1, TGF-β and PPAR-γ expression levels and MVD were analyzed with Pearson’s χ^2^ difference test. P<0.05 was considered to indicate a statistically significant difference.

## Results

**Figure 1:**
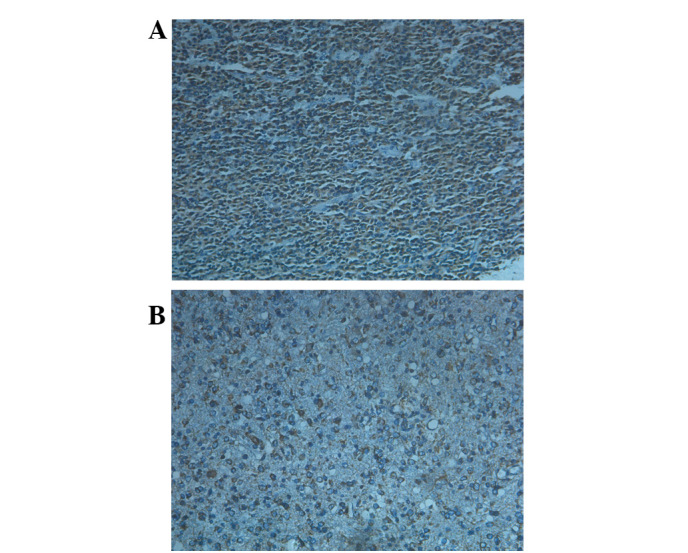
TSP-1 expression in low- and high-grade gliomas. TSP-1 (brown granular staining) was mainly expressed in the cytoplasm and extracellular matrix of the glioma. Expression was much higher in (A) low-grade than (B) high-grade tumors. SABC staining, magnification, ×200. TSP-1, thrombospondin-1; SABC, streptavidin-biotin complex.

**Figure 2:**
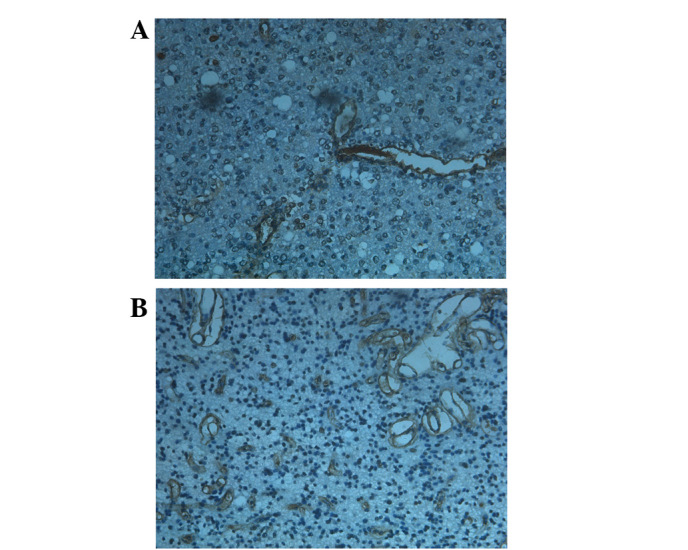
TGF-β expression in low- and high-grade gliomas. TGF-β (brown granular staining) was most strongly expressed in the cytoplasm of vascular endothelial cells. TGF-β expression was greater in (B) high-grade gliomas than in (A) low-grade tumors. SABC staining, magnification, ×200. TGF-β, transforming growth factor-β; SABC, streptavidin-biotin complex.

**Figure 3:**
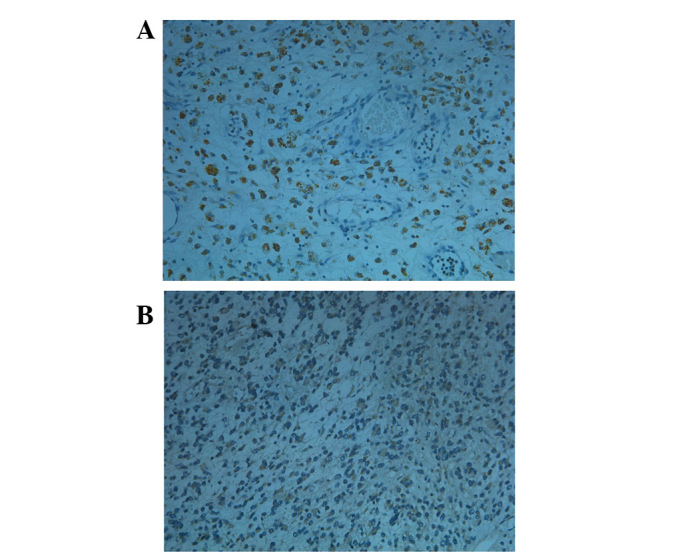
PPAR-γ expression in low- and high-grade gliomas. PPAR-γ (brown granular staining) was mainly expressed in the cytoplasm or nuclei of glioma cells. PPAR-γ expression was higher in (A) low-grade gliomas than (B) high-grade tumors. SABC staining, magnification, ×200. PPAR-γ, peroxisome proliferator-activated receptor-γ; SABC, streptavidin-biotin complex.

**Figure 4:**
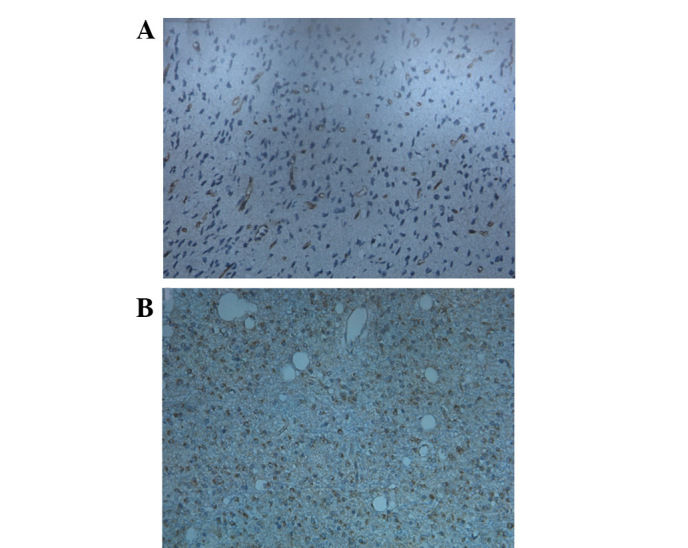
CD34 expression in low- and high-grade gliomas. CD34 immunoreactivity (brown) was mainly in the cytoplasm or membrane of glioma cells and is observed to be tubular, streak-like, comma-like and lumpy in staining shape. CD34 expression was much greater in (B) high-grade gliomas than in (A) low-grade tumors. SABC staining, magnification, ×200. SABC, streptavidin-biotin complex.

**Figure 5:**
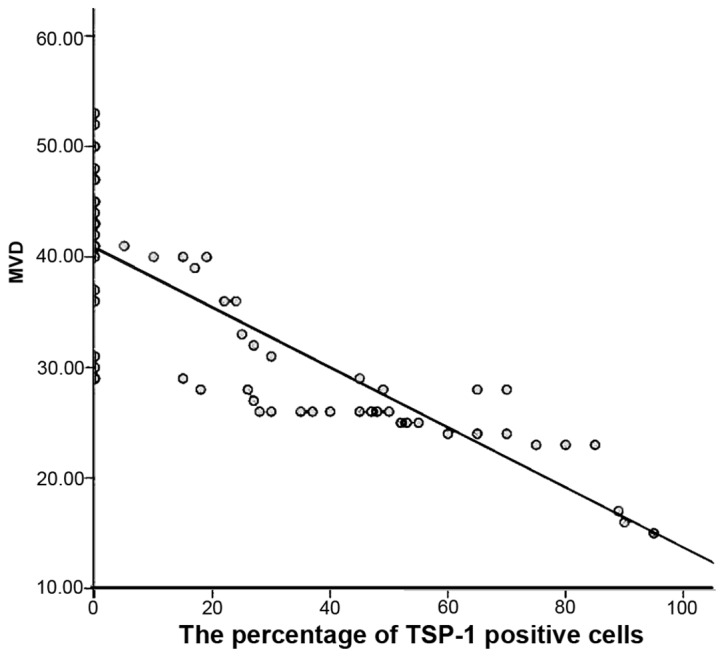
TSP-1 expression is negatively correlated with MVD (r=−0.61). TSP-1, thrombospondin-1; MVD, microvascular density.

**Figure 6:**
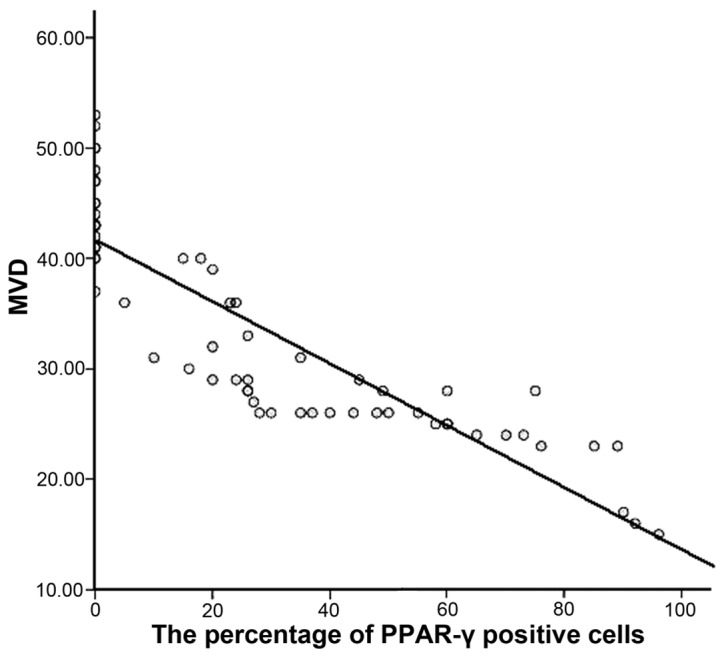
PPAR-γ expression is negatively correlated with MVD (r=−0.82). PPAR-γ, peroxisome proliferator-activated receptor-γ; MVD, microvascular density.

**Figure 7:**
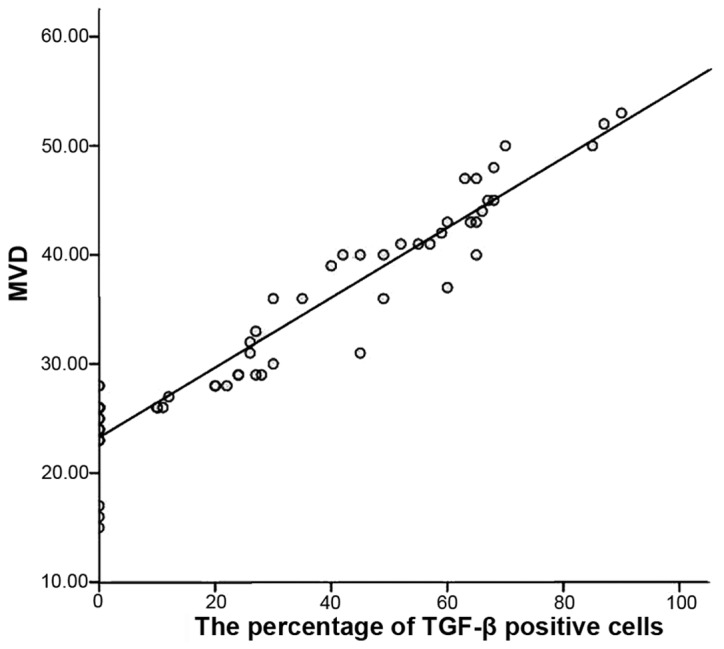
TGF-β expression is positively correlated with MVD (r=0.95). TGF-β, transforming growth factor-β; MVD, microvascular density.

TSP-1 was mainly expressed in the cytoplasm or intracellular space of the glioma cells and its expression was much higher in low-grade than in high-grade tumors (Fig. 1). TGF-β was predominantly expressed in the cytoplasm of vascular endothelial cells and the positive staining was greater in high-grade gliomas than in low-grade tumors (Fig. 2). PPAR-γ was largely cytoplasmic or nuclear and the expression was higher in low-grade gliomas than in high-grade tumors (Fig. 3). CD34 was mainly expressed in the cytoplasm or membrane of vascular endothelial cells and was often tubular, streak-like, comma-like and lumpy in staining shape, and it was much greater in high-grade gliomas than in low-grade tumors (Fig. 4). As presented in Table III, there were significant differences for the intense positive rate (χ^2^=16.4, 13.8, and 29.7, respectively) and total positive rate (χ^2^=33.6, 11.2, and 29.0, respectively) of PPAR-γ, TSP-1 and TGF-β, between low- and high-grade gliomas. The MVD in the high-grade glioma group (45±6.2 vessels/field) was significantly higher than that in the low-grade glioma group (28±7.2 vessels/field) (t=2.17). Immunoreactivity for TSP-1 (r=−0.61; Fig. 5) and PPAR-γ (r=−0.82; Fig. 6) was negatively correlated with the MVD, whilst TGF-β expression levels were positively correlated with the MVD (r=0.95; Fig. 7).

## Discussion

TSP-1 is reportedly involved in tumor angiogenesis by mediating endothelial cell migration and apoptosis, and regulating vascular endothelial growth factor (VEGF) expression (11–14), but these functions are controversial. Previous studies have demonstrated that TSP-1 inhibits tumor angiogenesis and invasion in melanoma and breast, prostate, and pancreatic cancers (3–6,15–17). However, Elpek *et al*(18) observed that TSP-1 promotes angiogenesis during chronic liver injury and did not identify a correlation between TSP-1 expression and MVD in pituitary tumors (19). In the present study, it was demonstrated that TSP-1 is negatively correlated with the MVD in the gliomas, and TSP-1 immunoreactivity decreases with increasing tumor grade, suggesting that TSP-1 may inhibit tumor angiogenesis and progression in gliomas. Fontana *et al* reported that TSP-1 inhibits tumor angiogenesis in the early stages of tumor growth and induces local hypoxia to produce greater quantities of VEGF, which promotes angiogenesis and inhibits TSP-1 expression (20).

TSP-1 activates latent TGF-β, thus increasing TGF-β expression to affect the biological behavior of tumors. In malignant tumors, TGF-β promotes tumor angiogenesis, immune escaping and metastasis, but it has opposite effects in normal epithelial cells during early tumor stages (1,12,13,21). The effect that TGF-β exerts on tumor progression may ultimately depend on the tumor microenvironment (13,14,22–24). The results of the present study demonstrated that TGF-β is mainly expressed in the surrounding blood vessels, and its expression is positively correlated with the MVD, particularly in the high-grade gliomas. These findings suggest that TGF-β may promote angiogenesis in gliomas. The comprehensive effect of TSP-1 and TGF-β in tumors may be influenced by the balance between anti-angiogenic and invasive factors. Further studies are required to investigate the interaction of TSP-1 and TGF-β in regulating glioma angiogenesis.

The possible tumor-suppressive effect of PPAR-γ remains controversial. PPAR-γ agonists applied *in vitro* are able to inhibit tumor cell proliferation and decrease the expression of extracellular matrix proteins, such as type I collagen and fibronectin (8,9). PPAR-γ agonists inhibit tumor angiogenesis through different mechanisms. For example, they may induce and activate hepatocyte growth factor, which activates C-methionine receptors and upregulates Smad transcriptional repressor expression, thus blocking the Smad pathway required for TGF-β nuclear translocation (25). PPAR-γ agonists have also been shown to block the epithelial-mesenchymal transition (EMT), which inhibits tumor metastasis by inhibiting the Smad pathway (7). In addition, PPAR-γ agonists upregulate the expression of CD36, a TSP-1 receptor, which in turn promotes TSP-1 expression and inhibits tumor angiogenesis (26). The present study identified that PPAR-γ expression was significantly different between the low- and high-grade gliomas and was negatively correlated with the MVD, suggesting that PPAR-γ may inhibit angiogenesis in gliomas. In addition, PPAR-γ expression in the gliomas was negatively correlated with TGF-β (r=−0.38, P=0.002), but positively correlated with TSP-1 (r=0.37, P=0.003) (data not shown), suggesting that PPAR-γ inhibits angiogenesis by regulating TSP-1 and TGF-β expression.

In the present study, we observed that the expression levels of TSP-1, PPAR-γ, and TGF-β correlated with the glioma grades. Furthermore, TSP-1 and PPAR-γ expression levels negatively correlated with MVD, while TGF-β expression levels positively correlated with MVD. Collectively, these results suggest that TSP-1 and PPAR-γ expression levels are closely correlated with angiogenesis in gliomas and may exert a synergistic effect, which may provide potential therapeutic targets for glioma therapy. TSP-1 and PPAR-γ expression in gliomas may also serve as indicators for tumor malignancy and prognosis, whereas TGF-β promotes angiogenesis during glioma progression and its expression is correlated with the degree of malignancy. Therefore, TGF-β expression in gliomas may serve as an indicator for tumor malignancy.

## Figures and Tables

**Table I:** Patient clinical data.

Variable	Low-grade group (n)	High-grade group (n)
Cases	51	48
Age		
≥40 years	20	25
<40 years	31	23
Gender		
Male	30	24
Female	21	24
Tumor site		
Supratentorial	44	32
Subtentorial	7	16
Operative method		
Total resection	40	34
Subtotal resection	11	14

**Table II:** Detection results of MVD in low- and high-grade glioma.

Groups	Number of cases	MVD (vessels/field)			t

		Minimum	Maximum	Mean	
Low-grade	51	15	40	28±7.2	2.17
High-grade	48	28	53	45±6.2	
Total	99	15	53	33±9.5	

MVD, microvascular density.

**Table III:** TSP-1, TGF-β and PPAR-γ expression levels by glioma grade.

Indicators	Groups	Cases (n)	Negative (−)	Mild positive (+)	Positive (++)	Intense positive (+++)	Total positive rate (%)	P_1_-value (χ^2^)	Intense positive rate (%)	P_2_-value (χ^2^)
TSP-1	Low-grade	51	11	3	18	19	78.4	0.00^a^ (11.2)	37.3	0.00^a^ (13.8)
	High-grade	48	26	13	6	3	45.8		6.3	
TGF-β	Low-grade	51	29	11	8	3	43.1	0.00^a^ (29.0)	5.9	0.00^a^ (29.7)
	High-grade	48	3	3	15	27	93.8		56.3	
PPAR-γ	Low-grade	51	3	8	19	21	94.1	0.00^a^ (33.6)	41.2	0.00^a^ (16.4)
	High-grade	48	29	8	8	3	39.6		6.3	

a P<0.01 vs. high-grade group.

TSP-1, thrombospondin-1; TGF-β, transforming growth factor-β; PPAR-γ, peroxisome proliferator-activated receptor-γ.
